# Supplementary figures and images for: Visual perception of liquids: Insights from deep neural networks
Source: PLoS Comput Biol. 2020 Aug 19;16(8):e1008018. doi: 10.1371/journal.pcbi.1008018 (PMC7437867; doi:10.1371/journal.pcbi.1008018)

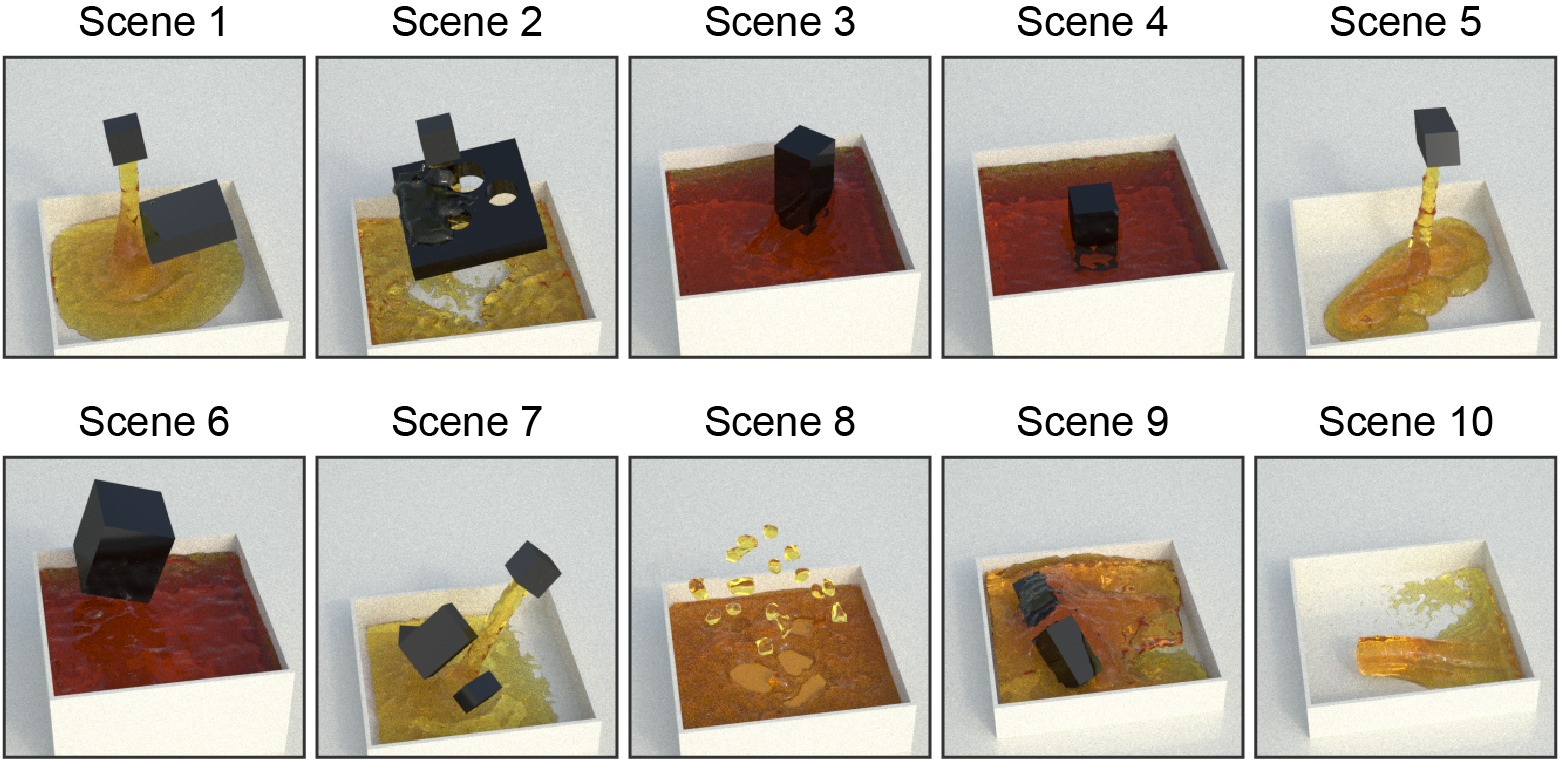

Supplement: S3 Fig — In this case all optical and physical parameters are held constant across stimuli except for the changes in viscosity making the images much more similar. (TIF) [file pcbi.1008018.s006.tif]
