# Supplementary material for: Visual perception of liquids: Insights from deep neural networks
Source: PLoS Comput Biol. 2020 Aug 19;16(8):e1008018. doi: 10.1371/journal.pcbi.1008018 (PMC7437867; doi:10.1371/journal.pcbi.1008018)
Supplement: Attachment — Submitted filename: ReviewersResponses.pdf [file pcbi.1008018.s009.pdf]

Responses to reviewers

We would like to thank the reviewers for their comments, which we think have greatly improved this manuscript in both scientific value and clarity. We adopted many of the reviewers' recommendations and here provide detailed responses to each comment.

Reviewer's Responses to Questions

Comments to the Authors:
Please note here if the review is uploaded as an attachment.

Reviewer #1: This manuscript presents an initial and exploratory look at DNNs as models of viscosity perception. The authors join a growing number of researchers in the field in trying to model perceptual computation using DNNs. The authors collected human judgments of viscosity in response to short video clips of simulated liquids. They chose a single DNN architecture to train on the same task and compared its errors to human errors, finding that it accounted for a reasonable amount of the variance. Next, the authors conducted a series of analyses to understand various aspects of the solution(s) found by the model. They conducted representational-similarity analyses to compare unit activations to various hand-engineered features in order to understand the kinds of information that the network uses. As is par for the course when training DNNs as models of perception, the principle conclusion seems to be that neural networks learn complex, difficult-to-interpret functions that don't map nicely onto any known set of hand-engineered features, but they are currently the best models we have (or at least the best image-computable models), so more work needs to be done. The manuscript represents a promising start, but needs some improvements to the writing, and might be stronger and more interesting with a few additional analyses.

My critiques fall into a few categories:

1. In various places, the interpretation of the network analysis results seemed to be on shaky ground.

i) For example, starting line 474:
"This is further corroborated by the good transfer learning performance in which the final layers were retrained for a different task. It supports the idea that earlier layers converge on task-independent image representations: a basic toolbox of filters that can be applied for a wide range of visual tasks and are similar to the earlier processing stages in our visual cortex." Are the authors trying to state that a general property of DNNs is to get some amount of task-generalization for free, and therefore this validates DNNs generally as models of perception? If so, this argument needs a little work.

We have reworded this text in the manuscript as follows:

Since representing these additional characteristics neither helps nor hinders task performance, the sensitivity to seemingly redundant features is likely a by-product of having excess representational capacity in the network. This suggests caution is necessary in drawing conclusions about biological representations from neural network models, as quite different representations at a given stage of the network can yield near identical decisions by the system as a whole. These findings demonstrate that early layer features not only contain powerful processing capabilities for the given task, but as a by-product are descriptive enough to provide a foundation for inferring a rich variety of other high-level scene factors. This is further corroborated by the good transfer learning performance in which only the final layers were retrained for a different task. It supports the idea that earlier layers have the tendency to converge on task-invariant image representations: a basic toolbox of filters that dissect visual information for further processing in a wide range of visual tasks and are similar to the earlier processing stages in our visual cortex [35–40]. We speculate that this is not a universal characteristic of DNNs, but the result of learning a challenging task on a rich training set with a network that has greater than necessary capacity. This requires further research and leads to the intriguing speculation that cortical visual representations might be as much the result of the *number of cells* in the different brain regions, as the specific task(s) the visual system learns.

We want to make the point that our results support the idea that many of the early layer representation are building blocks that can be used for many visual tasks. We would not argue it is a general property of DNNs, but more a result of learning a challenging task on a rich training set with a network that has greater than necessary capacity. We are certainly not the only authors to observe the general-purpose utility of features in early layers of neural networks, and indeed the very concept of transfer training rests on this assumption.

I might recommend something like the following framing to improve flow: The DNNs we trained can perform viscosity judgments about as well as humans, and they are argued to be somewhat plausible models of the perceptual system. Therefore, we reasoned that we could explore the networks' learned representations to gain

some insight into the realm of possibilities for analogous computations in the brain. To the extent that human's representations are learned wrt similar objectives and constrained in similar ways to the DNNs we trained on this task, our analyses suggest it is unlikely that human viscosity judgments rely exclusively on a set of easy-to-identify features or cues.

We thank the reviewer for this outline. We've made numerous tweaks to the manuscript to support a similar structure.

The authors might also consider more closely connecting their own work to similar work in other areas of perception (see e.g. Bates & Jacobs (2019) for one review of DNNs as models of behavioral data). I see a handful things are cited, but from the text, I didn't get a strong impression that this work is well-situated within previous related works.

We have added the following references to link our work to related research using DNNs to model behavioural data:

43. Dubey R, Peterson J, Khosla A, Yang M-H, Ghanem B. What Makes an Object Memorable? In: The IEEE International Conference on Computer Vision (ICCV). 2015.
44. Kubilius J, Bracci S, de Beeck HPO. Deep neural networks as a computational model for human shape sensitivity. PLoS Comput Biol. 2016;12(4):e1004896.
45. Peterson JC, Abbott JT, Griffiths TL. Adapting deep network features to capture psychological representations. ArXiv Prepr ArXiv160802164. 2016;
46. Kummerer M, Wallis TS, Gatys LA, Bethge M. Understanding low-and high-level contributions to fixation prediction. In: Proceedings of the IEEE International Conference on Computer Vision. 2017. p. 4789–4798.
47. Geirhos R, Janssen DH, Schütt HH, Rauber J, Bethge M, Wichmann FA. Comparing deep neural networks against humans: object recognition when the signal gets weaker. ArXiv Prepr ArXiv170606969. 2017;
48. Greene MR, Hansen BC. Shared spatiotemporal category representations in biological and artificial deep neural networks. PLoS Comput Biol. 2018;14(7):e1006327.
49. Tamura H, Prokott KE, Fleming RW. Distinguishing mirror from glass: A'big data'approach to material perception. ArXiv Prepr ArXiv190301671. 2019;
50. Kindel WF, Christensen ED, Zylberberg J. Using deep learning to probe the neural code for images in primary visual cortex. J Vis. 2019;19(4):29–29.
51. Storrs KR, Fleming RW. Unsupervised Learning Predicts Human Perception and Misperception of Specular Surface Reflectance. bioRxiv. 2020;
52. Lake BM, Ullman TD, Tenenbaum JB, Gershman SJ. Building machines that learn and think like people. Behav Brain Sci. 2017;40.
53. Majaj NJ, Pelli DG. Deep learning—Using machine learning to study biological vision. J Vis. 2018;18(13):2–2.
54. Jacobs RA, Bates CJ. Comparing the visual representations and performance of humans and deep neural networks. Curr Dir Psychol Sci. 2019;28(1):34–39.

ii) Starting line 196: "this could suggest that our visual system make use only of the most salient cues in the dataset and ignores the subtler cues that are specific to the training set, which the network learns with greater training"
Or in other words, early learning has better out-of-sample generalization? This strikes me as a rather broad claim about DNNs, so I'd be surprised if there is no literature on it (e.g. in the field of 'multi-task learning').

This is not exactly the point we want to make. We have rephrased this section as follows:

This could suggest that the networks first latch onto the most salient and discriminable cues (i.e., early in training) and that the human visual system—in this specific task with relatively hard to interpret stimuli—relies on similar cues. Whereas human observers ignore or cannot discern the subtler cues that are specific to the training set, with greater training the network can pick up on these subtleties (i.e. red curve increasing in error over time, green curve decreasing in error over time).

We argue that the most discriminable cues, cues that show the largest variance across viscosity, are learned early in training. As training progresses the network singles out subtler cues that are more discriminative for this particular dataset or stimuli, cues the human observer maybe can't even discern. We also extended on this with an additional paragraph in the discussion section with additional references in support of this statement.

2. Some additional analyses may be warranted to better support the arguments presented, and the motivations for some analyses were unclear.
i) Why not compare other kinds of networks, trained differently or with different architectures? By exploring the space of DNNs a little, we might glean more insight into what elements are essential for capturing human errors. Is there anything specific to the chosen architecture that is important for capturing human errors? E.g., the authors might compare a network that is trained on still images, rather than video, as this eliminates temporal statistics. Are temporal statistics crucial to capture human judgments/errors?

In pilot work (see Figure R1), we did consider static as well as moving stimuli. However, as human observers performed rather poorly and inconsistently at viscosity estimation with static frames, we decided to concentrate on movies, and consequentially on networks that can process them.

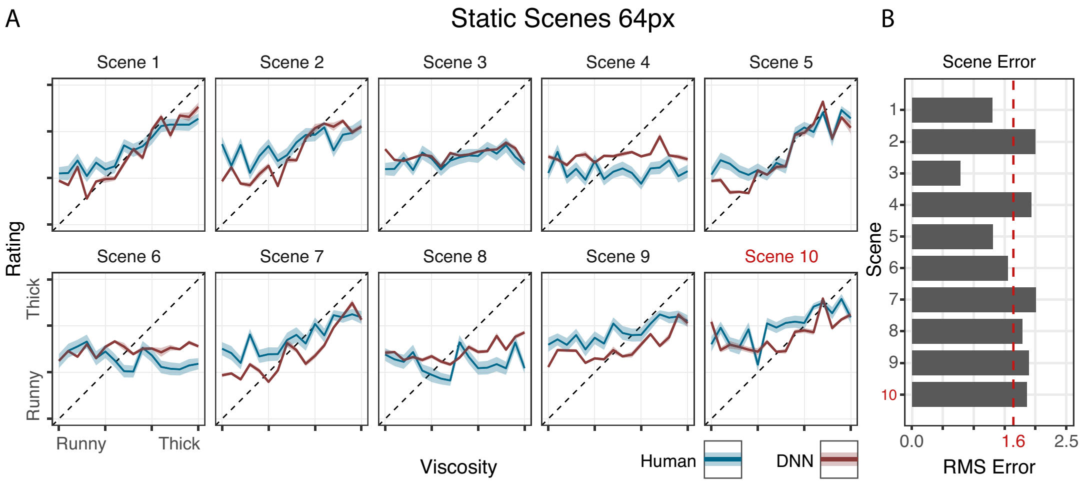

Figure R1: Perceived viscosity and neural network prediction for static stimuli.

The literature on video classification and regression networks is as not as mature as e.g. object classification in static images. Good datasets are scarcer, computational costs are high, there is a large variation of architectural design choices for video data, and the dimensionality of the spatial and temporal input varies widely making it harder to compare networks across visual problems. As a result, benchmarking is much less developed than with static object classification tasks.

The most mature spatial and temporal networks we have found focus on action classification in videos. We picked the two current best performing networks in this field of research (S3D-G and D3D) and applied them to our viscosity task. We didn't fully retrain the networks for this task. We gathered the neural activations generated by our liquids as input in the final stages of the networks before the prediction layers. We used these neural activations (12288 per stimulus) to train a gradient descent linear regression model (30 Epochs)—in practice a single unit fully connected layer with 12288 weights—to predict viscosity. We used the same test and training sets as with our own networks and picked the best model from 10 different instances. Figure R2 shows the results. We find that the networks actually perform well and seem to be quite powerful. The D3D network trained on the Kinetics 400 dataset has a 10% larger error. The networks architectures are quite different from the slow-fusion model we used, using 3D convolutions and inception modules that separate temporal and spatial information. Kinetics 600 dataset is a bigger dataset with 600 classification categories. What is interesting is that the difference between ground truth physical viscosity predictions and perceived viscosity predictions seems to be even larger with these networks. We have included these results in the discussion of the paper in support of our argumentation.

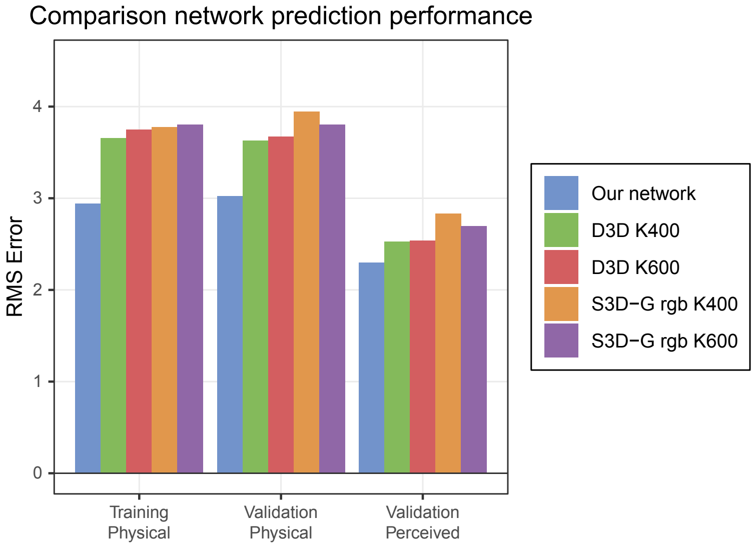

Figure R2: Comparison with alternative network architectures.

ii) The manuscript presented analyses of how well the hand-engineered features predicted network activations, but I was expecting there to also be some analysis of how well those same features predicted human responses. This seems like an easy sanity check to support the claim that human judgments of viscosity aren't easy to describe with known features. One could do a regression analysis, or perhaps RDM. Perhaps this was omitted because previous publications have already established it? Or some other obvious reason I'm missing?

There are many possible analyses to perform and we agree that this would be a nice addition in support of our claim that mid-level features are key. We have now performed this analysis and included it in the MS. To avoid bulking up the text too much, we report it in a single sentence:
Excluding the high-level predictors (viscosity and scene) we find that a RDM regression model of our image metrics only predict 2% of the perceived viscosity similarities ($R^2 = 0.02$, $F(1,13) = 376$, $p < .001$).
We used the Euclidean distance image metric RDMs to explain the perceived viscosity RDM (ground truth and scene ID were excluded). The performance is not good ($R^2 = 0.02$, $F(1,13) = 376$, $p < .001$), suggesting that the network is performing crucial higher-level computations that are not captured by our metrics. This is in line with our previous studies as well where similar metrics were not predictive of viscosity, especially across contexts.

iii) From line: 413: "To test this, we measured the ability of the network to classify the different scene classes by applying transfer learning on FC4 and subsequent layers". Why didn't you also test the 15-unit network to verify that it couldn't learn?

We agree that this was a logical addition, in our project the transfer learning analysis and squeezing FC4 representation were performed separately and therefore we overlooked this possibility. We kept the input weights of FC4-15 fixed. The output weights (150 new weights, FC4-15 to FC5-10) had to be retrained for the last prediction stages to translate the regression output to a softmax 10-class output. As expected, there is a drop in performance but it is not a very large drop. FC4-4096 achieves 88.63% classification accuracy (AUC = 0.993) and the FC4-15 layer yields 77.25% accuracy (AUC = 0.974). As with many of these analyses we picked the best result from 10 trained instances. We adjusted our arguments accordingly:
As expected, we find that the FC4-15 performs worse—although not terribly—for scene classification (77.25% accuracy, AUC = 0.974). These findings demonstrate the power of the features in the earlier layers which can be repurposed to perform different tasks.

But perhaps more importantly, the purpose of the whole enterprise (squeezing FC4) was unclear to me. Regarding this set of analyses, the authors say: "This demonstrates that when networks have capacity that exceeds the bare minimum required for the task, they may encode (i.e., retain, or fail to exclude) aspects of the stimuli that are not strictly necessary for task performance." Isn't this just a general property of DNNs? What does it tell us about people's biases or abilities?

This analysis doesn't tell us anything in particular about people's biases or abilities. This simply demonstrates the representational malleability of these networks and that caution is required drawing conclusions about the

encoded features we find in any given task for a DNN. It also implies that when using neural networks to reveal the most critical features in a specific visual task we really need to keep the number of network parameters to a bare minimum, or, otherwise, be explicitly aware that non-essential features are likely to be encoded in addition to the essential ones. In general, we could not find much discussion of network capacity as a key determinant of representations in the literature comparing DNNs to human sensory processes. We therefore think this is useful observation for vision scientists that are not that acquainted with neural networks and might not understand how architectural changes can radically change representations. To address the reviewer's concern, we now motivate the squeezing of FC4 more clearly, rather than simply describing what we did:
This raises an intriguing question about the nature of the representation in the final stages of the network: to what extent is the representation determined by the capacity of the network, rather than the demands of the objective? We reasoned that the tendency to represent factors that are seemingly unimportant to the task likely reflect excess representational capacity. To test this possibility we compressed the 4096-unit fully connected layer FC4 until the prediction performance started to decrease.

iv) How much does the Bayesian optimization of the hyperparameters help? This procedure was mentioned but I saw no discussion of how much it mattered for the results.

We agree that we did not give enough information about the effects of the Bayesian optimization. We predefined the search space for the Bayesian optimization. Our final network was closer to the minima of this search space than the maxima. To compare this, we performed full training sessions (i.e. 200 epochs) with the minima and maxima of the optimization space to quantify performance differences. As you can see in Figure R3, the best performance is achieved with our network properties. For fair comparison the hyperparameters (e.g. learning rate, momentum) were held constant. We now include information from this analysis in the manuscript:
The optimal settings were close to the minima of the space we searched, the maxima produced networks that performed 47% worse in terms of perceived viscosity predictions.

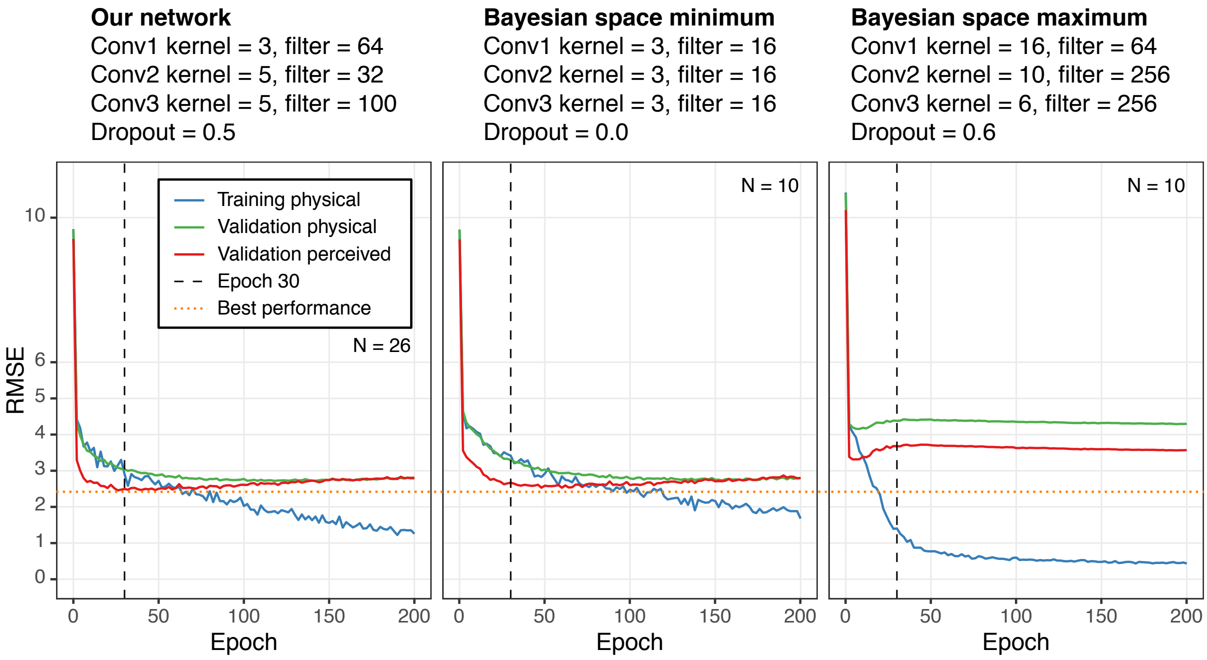

Figure R3

Figure R3 shows that in our predefined space, with our network architecture, Bayesian optimization indeed found the best parameters to achieve highest perceived viscosity performance. There is a performance difference of 47% between best and worst performance at epoch 30 (our network vs maximum space). Other properties change as well, such as overfitting (distance between blue and green), best training performance, and best performance peak (where the maximum space networks are around epoch 4 instead of 30). This suggests that our conclusions broadly generalize over a wide range of hyperparameter values, but that the Bayesian optimization does significantly improve our fit to human data.

v) Line 239: "This means that the same stimuli are encoded with different 'mid-level' image features".
The interpretation of why the second layer diverges on different re-trainings seems a little shaky, as they only tried out one network architecture, and only looked at FC layers, not conv layers. The relevance of this point to

the main findings is also unclear to me. At any rate, I might suggest the authors check out Kornblith, et. al (2019). It might be interesting to see what results from applying the new similarity metric proposed in that paper (which is easy to implement).

The second reviewer also suggested Centered Kernel Alignment analysis. We performed this analysis (see Figure R4) and indeed results are broadly in line with Kornblith's et. al. findings: we see a clear trend that the networks are very similar overall, becoming moderately more dissimilar as layer depth increases. Indeed, on the basis of this analysis, we have now removed claims about the differences in mid-level representations from the MS. For clarity we now only report CKA for the between-network comparisons.

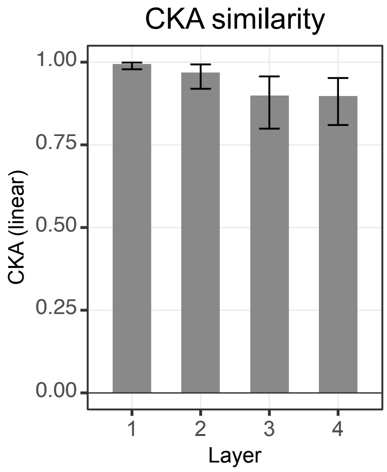

Figure R4: Between-network analysis now done with CKA instead of RSA. Error bars show 1st and 99th percentiles.

vi) Line 281: "To get a clearer impression of the unit-specific function we visualized the stimuli that minimally and maximally activate the unit". Why not also use gradient methods to find series of images that maximally activate the unit? Perhaps this would provide deeper insights. The authors dismissed this possibility in the discussion, but it wasn't clear whether they tried it or if they had sound reason to dismiss it a priori as unlikely to be useful. Did reference 22 specifically suggest for their architecture type that it is not useful?

Both Nguyen et. al. (mostly concentrating on static features) and the authors of the slow-fusion architecture Karpathy et. al. do not go into detail about visualizing features in slow-fusion networks. We looked into this and spent quite some time on making the network channels more interpretable. After this feedback we spend another iteration on activation maximization methods as newer implementations are now available. We still report the minima and maxima of the stimuli but included a section on the activation maximization as well. Because of the architecture design (i.e. small kernel sizes, fusion of channels, the mixture of temporal and color channels after layer 1), its results are quite abstract and not easy to interpret. See supplemental video S3 for the results.

vii) When the network was trained for greater than 30 epochs, on what kinds of simulations did the network diverge from human error? Can anything being discerned by visual inspection or other analysis?

This is a very intriguing suggestions, which we tried, but which unfortunately did not deliver very conclusive results. During training the network increasingly converges on the physical viscosity labels, but it also tends to overfit as well. To test out the reviewer's suggestion, we continued training network 78 (the standard network which we report throughout the paper). We looked at the stimuli whose prediction changed most, both in term of physical viscosity and perceived viscosity between the epoch 30 and epoch 200 networks, but visual inspection revealed no consistent patterns. We also looked at the tSNE representations and the main finding is that the representation tends to spreads out more with increased training. This is in line that with our previous observation that when training continues, the network tends to latch onto more dataset-specific cues, increasing the distance between stimuli in activation space. The largest differences are visible when we plot both epochs in one combined tSNE plot (Figure R5). Here we see that epoch 200 uses more of this space and the overall distance between stimuli is increased. However, as with visual inspection, we did not find anything consistent (e.g., the changes were not associated with a particular scene of viscosity level). As the analysis was ultimately rather inconclusive we decided not to include it in the MS.

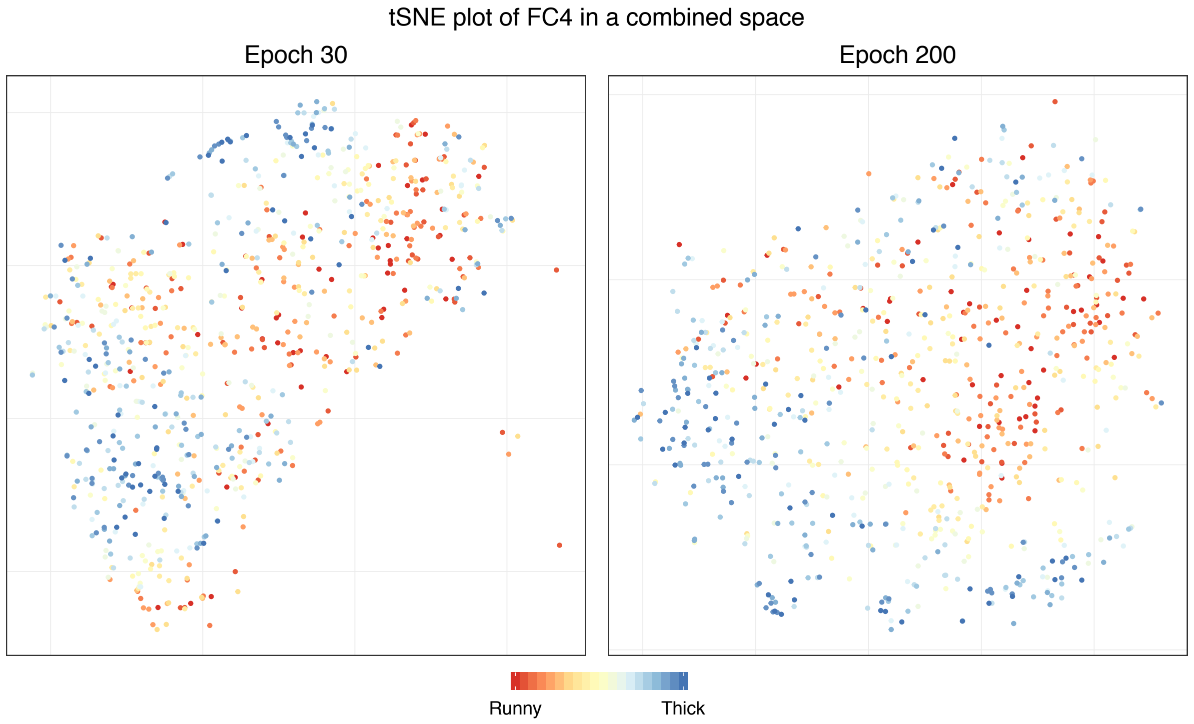

Figure R5

3. Misc.

i) In order to induce errors in participants, why didn't the authors choose to make the viscosity scale more fine-grained rather than make the images impoverished? I'm slightly concerned that this is less ecological, and therefore the results might not generalize as well. But maybe this is okay because it's like viewing it from farther away.

During piloting, we did also perform perceptual experiments with higher resolution images and didn't find any significant effects on the viscosity ratings. To keep the comparison between humans and model as close as possible, for the MS, we decided to test liquid simulations at the same spatial resolution for both human and machine observers. Computational costs were a major factor here. To run 20.000 particle simulations at high resolution and render 1 million images at higher sampling levels was not possible with our available infrastructure. It already took more than 2 months to generate the training set on one of the five scientific computing clusters available in Hessen, Germany. Training itself would also have been much costlier as well with higher resolution videos (or finer sampling of viscosity). In practice, we find that a 16-step scale is quite close to the discrimination threshold for much of the range. It is not clear how a finer-grained viscosity scale could make the results any more ecological: it would simply restrict our conclusions to a narrower range of viscosities.

ii) Line 212: "This overcomes the challenge of having sufficient labelled data to train directly on human judgments."
But training on human judgments would result in an empirical ("curve-fitting") model, which is an entirely different class of model with different associated goals. Therefore this statement may be a little misleading.

Many datasets today use labels generated by observers, imagenet included. Here we point out an alternative if ground truth labels are available. Generally, ground truth labels correlate quite closely with perceived labels so the difference in outcome from training with human vs. ground truth labels is not expected to be that large anyway. But in view of the reviewer's comment, we sharpened the text a little to clarify that the goals are not the same:
This partially overcomes the challenge of having sufficient labelled data to train directly on human judgments, and allows us to test the role of specific learning objectives and training sets in human performance.

iii) Why does Fig. 4 only use 26 networks, instead of 100?

The training of 26 networks showed that optimal performance is achieved at 30 epoch. Computationally it took too much time to run all 100 networks for 200 epochs, which was not necessary for our argumentation here. The dataset of 26 networks that ran for 200 epochs is a different set than the 100 networks trained for 30 epochs.

iv) Line 218: “In order to interpret the response patterns, we compare these neural activations with a set of predictors.” —> “In order to interpret the response patterns of individual units, …”

We do not only interpret response patterns of single units, but combine them on the layer level as well. Therefore, we retained the original sentence. We did make changes to this paragraph to make this clearer.

v) Typo, Line 290: “(D) A selection of the RSA correlations for the units closest to the centres of four of the clusters shown in Figure 6: (E)”. Should be Figure 7?

Thank you. The figures and their numbers have changed many times and this reference slipped through.

References:

Kornblith, S., Norouzi, M., Lee, H., & Hinton, G. (2019). Similarity of neural network representations revisited. arXiv preprint arXiv:1905.00414.

Jacobs, R. A., & Bates, C. J. (2019). Comparing the Visual Representations and Performance of Humans and Deep Neural Networks. Current Directions in Psychological Science, 28(1), 34-39.

Reviewer #2: Humans are extraordinarily good at making complex inferences about the world based on limited sensory information. One such remarkable ability is to infer intuitive physical properties such as the viscosity of flowing liquids. A model of how humans might use sensory information to make complex inferences has the potential to shed light on how humans might perform this same task, and this is the focus of this particular paper by van Assen and colleagues. To do this, the authors take the approach of training deep convolutional neural networks (DNNs) on a viscosity judgement task and then characterizing internals of this network using representational similarity analysis (RSA) and using other computer vision based descriptors like GIST etc.

The main claim of the paper is: 1) A particular DNN that can predict the behavior of human subjects. The secondary characterization of this DNN revealed units sensitive to certain to specific feature types apart from only viscosity and that it was possible to train a smaller network that achieves similar performance.

The main question raised in the paper is novel and of broad interest. However, the paper suffers several key conceptual issues with the network training paradigm, methods of comparing representations in deep networks and most strikingly, the lack of an alternative DNN model frameworks and an over-reliance on one specific method. Because of these issues, I am unable to recommend the paper for submission in its present form. I synthesize the four central issues with the paper below.

Main Issues:

1. The authors sample from a generative space to train networks which is a reasonable effort given the massive search space of possible videos. However, the authors do not seem to have fully utilized the strength of having this generative space. In particular, from the description in the Methods (Lines 579-580), the authors seem to have used many of the same video stimuli conditions to validate the models during training and to define an early-stopping criterion. Is this really correct? The reason this is confusing and inconsistent is because of Lines 144-147, and then the Lines 152 (“chose network 78 of 100”). What is the criterion of choosing a model and the criterion of defining a premature stop to the training. If the criterion is the match to human behavior, then it amounts to double-dipping, given that the central claim of the paper is the match of the DNN to behavior. This problem can be allayed by training the model (for example) on Scenes 1-8 (from Figure 1) but testing the match to behavior based on Scenes 9-10. The significant concerns about the training procedure and the potential double-dipping issue casts doubt on the authors’ main claims.

“…have used many of the same video stimuli conditions to validate the models during training and to define an early-stopping criterion”.
We already did exclude from our training set the 800 experimental stimuli as well as all stimuli from scene class 10 (i.e., almost exactly what the reviewer suggests). Each scene class spans many random conditions in all kinds of factors, so we have very diverse training and test sets to aid our tests of generalization. We agree that we could also have excluded e.g., certain viscosity values from training, introducing a gap in the trained scale but this would have quickly reduced the size of the training set. Across scenes, we introduced 77 randomly chosen physical parameters and 36 optical parameters, so we are highly confident that each stimulus shows unique and

diverse behavior. So, there is no double dipping. Comparable to research based on imagenet, we left out a certain percentage of stimuli of each class for validation during training, and left out one scene class altogether for even more explicit test of generalization. This is in line with what the reviewer suggests.

“What is the criterion of choosing a model and the criterion of defining a premature stop to the training. If the criterion is the match to human behavior, then it amounts to double-dipping, given that the central claim of the paper is the match of the DNN to behavior.”

All networks are only trained on physical viscosity labels, no learnable parameter was influenced by any human judgement. Stopping criteria for neural networks are often the point (in our case epoch 30) where validation performance doesn’t increase substantially anymore, in most cases this, and increasing differences between validation and test performance, identifies the pivotal point where the networks start to overfit (Figure 4). In this case this holds for validation of physical labels (on which the network was trained) and validation of perceived labels. We simply picked the point where the model performs best with our validation set (which consists of stimuli and corresponding perceived labels that the network has not encountered before).

This represents a generalization by itself as well as the model is learned for a different task than validated on, in this case predicting the physical viscosity and not perceived viscosity.

All 100 instances of networks were stopped after 30 epochs of training. All training conditions were exactly the same except for the random initialization and shuffled order of training stimuli. In a model with just short of 7 million learnable parameters some predictive variation occurs. This variation is small and we report this. Of the 100 networks we report the best predicting model in detail. However, we also demonstrate that there are many computational similarities between these networks (Figure S5).

“This problem can be allayed by training the model (for example) on Scenes 1-8 (from Figure 1) but testing the match to behavior based on Scenes 9-10.”

As mentioned above, we do already do almost exactly as the reviewer suggests and train the networks only on (in our case) scene 1-9 and test the match behavior of scene 10. We were concerned that this comment reflects a lack of clarity in the MS. Therefore, we sharpened the corresponding portions of the manuscript to make these differences clearer.

2. The authors rely heavily on one and only one model architecture in this paper. There are many DNN models now that can now be trained on videos and could therefore potentially be adapted to this particular task. While I don’t expect the authors to test ALL possible video based models out there, I do think that having at least a couple of additional models is necessary for any claim about this particular DNN model. Or do the authors think that there are many DNN models perform the same task. The writing and framework of the paper suggests one particular DNN framework as a model for this ability. Also, how constrained is the space of models given only one metric of matching behavior?

This point was raised by reviewer 1 as well and we would like to refer to our response there (see Figure R2, showing comparison to two leading video-based action classification models). We find that other much more extensively trained networks on action recognition perform 10% and upwards worse in this task using a tailored decoder for the 12288 unit responses of these networks. We included a section with these results in the manuscript.

3. How to compare DNN and other representations? This is an important question and while there are many methods, it has recently been brought to notice that representational similarity analysis (used in the paper) has particular flaws that make it less suitable for this purpose. I would urge the authors to revisit this question using updated tools (eg. Centered kernel Analysis) from statistical physics which have been used to probe this exact question in deep networks. See here for more details:

Similarity of Neural Network Representations Revisited (Korblith et al., 2019) https://arxiv.org/abs/1905.00414

This same concern was mentioned by reviewer 1 as well. We performed CKA for the between network comparisons and indeed find similarity differences compared to RSA (Figure R4). We revised our results using this particular analysis which are in support of arguments made by Kornblith et al.

4. From the analyses in Figures 5-7, it seems that the networks extract several low-level spatial and temporal statistics of the videos. While this is interesting, it undermines the argument that viscosity detection is a complex task that does not rely on low-level cues. Another way to look at this would be to train linear decoders from

specific layers to extract the viscosity levels. Have the reviewers tried this? How good are early layers of the network at performing the same task?

To the best of our knowledge, we never claim that low-level cues are not relied upon, especially since we demonstrate the networks use many of these low-level features: they form the building blocks for many mid-level concepts which we think are crucial. Yet at the same time, low-level cues themselves are not sufficient. A multiple linear regression using the image metric RDMs of the RSA analysis to explain perceived viscosity does performs poorly ($R^2 = 0.02$, $F(1,13) = 376$, $p < .001$). This would further support our argumentation around the unidentified category of units we find in layer 3 that are important higher-level units for the viscosity predictions and do not correlate with any of our lower-level metrics.
Furthermore, we also performed the linear decoding analysis as suggested by the reviewer. The table below shows the results of different metrics specifying the performance at each layer. The weights to the linear decoder need to be trained in each case (we used Bayesian optimization to find the optimal hyperparameters for each layer, especially the learning rate varies much across layers). We then performed 10 iterations of 10 epochs and take the average (N=10). The normalized RMSE rescales the minima and maxima to viscosity values of our original scale i.e. 1-16, especially for layer one we get predictions out of our original viscosity range due to the fixed activation amplitudes. We see that performance in both error and correlation increases as we go deeper, although the differences of the output after layer 3 and the full network are quite small (contribution of FC4).

	Layer 1	Layer 2	Layer 3	Full Network
RMSE	9.21	2.57	2.37	2.30
Normalized RMSE	3.62	2.48	2.29	2.42
Correlation (Pearson)	0.27	0.72	0.76	0.78

We made changes based on these results in the manuscript to emphasize the importance of each layer more precisely:
We trained linear decoders at each layer to predict perceived viscosity and find trends in line with the correlation of perceived viscosity shown by RSA. Already by ReLU2, viscosity prediction error is reduced to RMSE of 2.57—a 12% larger error than the full network (RMSE = 2.30). ReLU3 encodes enough information to perform at the same level as ReLU4 in terms of prediction error.
We thank the reviewer for the suggestion and believe this addresses it.
