# Supplementary material for: Visual perception of liquids: Insights from deep neural networks
Source: PLoS Comput Biol. 2020 Aug 19;16(8):e1008018. doi: 10.1371/journal.pcbi.1008018 (PMC7437867; doi:10.1371/journal.pcbi.1008018)
Supplement: Attachment — Submitted filename: ReviewersResponses.pdf [file pcbi.1008018.s010.pdf]

Responses to reviewers

We would like to thank the reviewers for their comments, which we think have greatly improved this manuscript in both scientific value and clarity. We adopted many of the reviewers' recommendations and here provide detailed responses to each comment.

Reviewer's Responses to Questions

Comments to the Authors:
Please note here if the review is uploaded as an attachment.

Reviewer #1: The authors made various additions in response to comments. The most consequential of these was to train additional network architectures on the task to compare to the original choice. I think this addition has improved the manuscript. I also think the multiple linear regression analysis between the hand-picked features and viscosity was helpful, and should be presented more prominently, as it supports the authors' central argument. Similarly, I think the linear decoding of viscosity from earlier layers provided good results that bolster the central argument, and yet this felt buried.
In line with the reviewer's third comment, we further emphasized the arguments and wording in support of our main findings. In particular, we have increased the prominence of these analyses by adding a few sentences to elaborate on them in the Results section, and in the case of the multiple linear regression, we also mention the finding in the new section of the introduction (see below for more details).

However, I still find the authors' interpretation of the crucial role for early stopping to be highly speculative, and hand-wave-y. For example, what is precisely meant by "salient cues"? How could this be operationalized in a technical way? There are other possibilities to think about. For instance, if the network were somehow trained on a larger dataset that is more ecological, would we find that it could be trained to convergence and still predict human responses well? In other words, is the choice of dataset driving the early stopping result, or is it some suboptimality or information processing capacity limit in humans? I would encourage the authors to either remove the speculation entirely, or flesh it out more. (I didn't find the brief discussion involving refs 32 and 33 to be very convincing.)
We certainly want to make sure we clearly distinguish between our concrete results and speculative interpretations. To address this concern, we have now removed the speculation entirely from the results section (we report the result but do not comment on it at that stage). However, we do think the finding is interesting enough to warrant further discussion, even if some of the ideas or possibilities considered are speculations—as long as they are clearly labelled to the reader as such. Accordingly, we have now modified the text in the Discussion section to flesh it out, following the reviewer's advice.
In particular, we have revised this paragraph as follows:

Our networks were trained for only 30 epochs, which is a relatively short time. We found that after epoch 30 the perceived viscosity predictions worsened and the networks increasingly started to overfit (**Fig 4**). This is further demonstrated by the increasing difference between training error with physical viscosity labels and validation error with physical viscosity labels after epoch 30. It is interesting to speculate about the causes and implications of this finding of a U-shaped approximation to human performance as a function of training. For example, it could be an artefact of the training set. While humans learn to see from diverse and naturalistic stimuli, the models considered here were trained exclusively on computer simulations of liquids. It could be that with more varied, larger or natural training data, the approximation to human performance would continue to improve with further training on the physical estimation task (i.e., no U-shaped approximation to human performance would be observed). However, another possibility is that the cues used by human observers are those that network also tends to learn first. It could be these cues are the most discriminable or most robust [32–34] cues of the dataset. As training continues, the network continues to improve at the physical viscosity estimation objective, possibly by learning more subtle cues specific to the dataset which the human visual system cannot discern at all or is less sensitive to. There is indeed some support for this speculation. Other studies that look into the early phase of neural network learning find critical learning periods similar to biological networks [35,36]. Evidence suggests that neural connectivity is broadly settled in a memory formation phase early in training, after which neuroplasticity decreases and only much smaller changes occur by reorganizing or forgetting less predictive weights [37,38]. This makes the early phase (< Epoch 10) an especially critical period where dominant qualities of the dataset are encoded. In our case this period is defined by an especially large drop in perceived viscosity error. This is in line with our speculation that the most discernible cues which are encoded in early training align particularly well with the perceived viscosity cues used by humans.

In general, I would also encourage the authors to spend a little more time with the writing in order to increase the impact of the work. The manuscript seems to lose focus and structure at times, which makes it hard to follow and thoroughly evaluate. To me, the most solid takeaway is that the model finds non-trivial features that support viscosity judgment, which are hard to intuit but essential both for doing the task and explaining human judgments. But this point came through a little muddled.

The MS certainly covers quite a few findings and analyses, and we agree that it needed additional work to clarify the narrative and guide the reader to the most important results. We have made numerous changes throughout the MS to improve this. The most significant change is that we have now added four new paragraphs to the end of the intro to: (1) provide a clear outline for the rest of the MS, (2) explain the logic of the analyses we performed and (3) summarize and emphasize the main findings. We believe this already significantly increases the readability and potential impact of the work. Additionally, we have added new subheadings to break the MS into smaller chunks, and at the beginning of each section of the results, we give a clearer rationale for what follows, to keep the text focussed. Finally, we have also streamlined the discussion section and highlighted the most important findings. Together, we believe these changes significantly improve the MS.

A misc. comment: I didn't see anywhere detailing the methods for "activation maximization" (or even so much as defining what it was).
Thanks for spotting this. We used a fairly standard approach to activation maximization, and we have now added a paragraph in the Methods section to explain the procedures in detail.

Reviewer #2: In the paper van Assen and colleagues train deep convolutional neural networks (DNNs) based models on a viscosity judgement task and characterize the internals of this network using representational similarity analysis (RSA) and other computer vision based descriptors like GIST.

I raised some important issues related to the exact way the models were trained (and concerns about potential double-dipping) and over-reliance on a single model architecture for their inference.
The authors have now addressed these issues by clarifying the model training regimen, and by testing a couple other leading video based models. These results, I feel, substantively improve the manuscript. I suggest that the authors emphasize that the models were trained on physical rather than perceived viscosity to make sure that readers do not miss this point.
We are happy our explanation cleared up the point of double dipping. As suggested by the reviewer we have further emphasized the differences between physical and perceived viscosity labels throughout the MS.

Going further, the authors also apply more statistically complex methods to compare network representations and show that low-level cues extracted from the videos by themselves not sufficient. This directly addresses the point about low-level features.

Taken together, I feel that the authors have satisfactorily addressed my concerns. I congratulate the authors on their interesting finding and recommend publication in its current form.
